# Sub-Micropillar Spacing Modulates the Spatial Arrangement of Mouse MC3T3-E1 Osteoblastic Cells

**DOI:** 10.3390/nano9121701

**Published:** 2019-11-28

**Authors:** Benedetta Ghezzi, Paola Lagonegro, Naoki Fukata, Ludovica Parisi, Davide Calestani, Carlo Galli, Giancarlo Salviati, Guido M. Macaluso, Francesca Rossi

**Affiliations:** 1 Centro Universitario di Odontoiatria, Università di Parma, Via Gramsci 14, 43126 Parma, Italy; ludovica.parisi@unipr.it (L.P.); guidomaria.macaluso@unipr.it (G.M.M.); 2 Dipartimento di Medicina e Chirurgia, Università di Parma, Via Gramsci 14, 43126 Parma, Italy; carlo.galli1@unipr.it; 3 ISMAC-CNR, Institute for macromolecular studies, Via Corti, 12, 20133 Milano, Italy; paola.lagonegro@gmail.com; 4 IMEM-CNR, Institute of Materials for Electronics and Magnetism, Parco Area delle Scienze, 37/A, 43124 Parma, Italy; davide.calestani@imem.cnr.it (D.C.); giancarlo.salviati@cnr.it (G.S.); francesca.rossi@imem.cnr.it (F.R.); 5 International Center for Materials Nanoarchitectonics, National Institute for Materials Science, 1-1 Namiki, Tsukuba 305-0044, Japan; FUKATA.Naoki@nims.go.jp; 6 Institute of Applied Physics, University of Tsukuba, 1-1-1 Tennodai, Tsukuba 305-8573, Japan; 7 Labör für Orale Molekularbiologie, Klinik für Kieferorthopädie, Zahnmedizinische Klinik, Universität Bern, Freiburgstrasse 7, 3008 Bern, Switzerland

**Keywords:** pillar, scaffold, osteoblasts, regenerative medicine

## Abstract

Surface topography is one of the main factors controlling cell responses on implanted devices and a proper definition of the characteristics that optimize cell behavior may be crucial to improve the clinical performances of these implants. Substrate geometry is known to affect cell shape, as cells try to optimize their adhesion by adapting to the irregularities beneath, and this in turn profoundly affects their activity. In the present study, we cultured murine calvaria MC3T3-E1 cells on surfaces with pillars arranged as hexagons with two different spacings and observed their morphology during adhesion and growth. Cells on these highly ordered substrates attached and proliferated effectively, showing a marked preference for minimizing the inter-pillar distance, by following specific pathways across adjacent pillars and displaying consistent morphological modules. Moreover, cell behavior appeared to follow tightly controlled patterns of extracellular protein secretion, which preceded and matched cells and, on a sub-cellular level, cytoplasmic orientation. Taken together, these results outline the close integration of surface features, extracellular proteins alignment and cell arrangement, and provide clues on how to control and direct cell spatial order and cell morphology by simply acting on inter-pillar spacing.

## 1. Introduction

Implanted devices require an adequate tissue-material interface to properly function [1], and it is therefore not surprising that a great deal of effort has been made to investigate and implement surface topographies capable of directing cell behavior and thus affecting the formation of vital tissue connecting the organism and the implant [2]. Endosseous implants in particular have a long history of being developed along these lines of thought [3]. Most endosseous devices are provided with roughened surfaces, which have been shown to promote the differentiation of osteoblasts and therefore to a phenotype that is capable of depositing new bone [4,5]. Starting from the assumption that macroscopic irregularities would improve implant retention by mechanical interlocking, research studies have progressively shown that tissues and cells therein can discriminate the presence of roughness on a micron and sub-micron scale and that the geometry and pattern of surface irregularities affect cell behavior [6,7]. Furthermore, surface wettability [8], surface charges, protein adsorption [9,10,11], and material stiffness [12] can all compound on the terminal biological effect of the material, thus making it very difficult to isolate the features that can regulate cell behavior and how these can be tuned as desired.

The first relevant step to establish an effective cell–material interface is cell adhesion [13]. How cells attach to a surface then dictates cell shape [14]. Cell morphology has then been closely associated to transcription of programs for cell differentiation [6]. Cell adhesion is at the center of cell activity, for adhering cells, such as osteoblasts. The formation of focal adhesions, the main attachment mediators, allows the organization of cell cytoskeleton and its mechanical activation, which in turn shapes how focal adhesions align in the cell [15,16]. Actin microfilaments are mainly responsible for the generation of tensile forces in the cytoskeleton [17], thanks to the combined activity of actin and myosin, and cell tension is required for the activation of mechanically-regulated pathways, such as YAP/TAZ signaling [18], and, for nuclear deformation, which has been shown to affect the transcriptional activity of specific chromatin domains, which, e.g., control cell differentiation [19]. To better understand cell morphology on biomaterials, new instruments have been developed, such as the Focused Ion Beam Microscope, which combines a Scanning Electron Microscope with a Gallium ion beam that can cut through samples, through minimal manipulation, and allow for close examination of their conformation [20]. The present study relied on such microscopy tools to investigate how hexagonal micro-patterned substrates affect the behavior of murine calvaria MC3T3 osteoblastic cells, a well-known model for bone cells that is capable of mimicking many physiological features of normal osteoblasts [21].

## 2. Materials and Methods

**Surface preparation.** Arrays of pillars were formed by nanoimprint lithography and Bosch etching using *p-type* (100) Si substrates cleaned with acetone (Sigma-Aldrich, St. Louis, MI, USA), ethanol (Sigma-Aldrich, St. Louis, MI, USA) and deionized water (DI-water). A 30 nm thick Cr layer was patterned on the *p-type* Si substrates using UV-imprint lithography. After lift-off processes, the Bosch process, using SF_6_ and C_4_F_8_ plasma with radio frequencies (RF) power of 100 W, was used for deeply etching the Si substrates to form the pillar array structure [22,23]. Two hexagonal arrays of pillars were produced, differing in the horizontal distance (3.6 and 4.0 microns, respectively) between neighbor hexagons; pillar diameter was 600 nm and their height was 900 nm.

**Cell cultures.** Osteoblastic MC3T3-E1 cells from mouse calvaria were obtained from the American Type Culture Collection (LGC Standards S.r.L., Sesto S.Giovanni, Milan, Italy) and cultured in Alpha-MEM (*α*-MEM, Thermo Fisher Scientific, Carlsbad, CA, USA) supplemented with 10% Fetal Bovine Serum (FBS, Thermo Fisher Scientific, Carlsbad, CA, USA), 1% L-Glutammine (Thermo Fisher Scientific, Carlsbad, CA, USA) and 1% Penicillin and Streptomycin (Penstrep, Sigma Aldrich, St. Louis, MI, USA). For the experiments, nanostructered silicon substrates (size 0.5 cm × 0.5 cm) were placed in 24-well plates and 2 × 10^4^ cells were seeded on substrates in a final volume of 1 mL, to perform viability assays, morphology observation and immunofluorescence.

To inhibit microtubule polymerization on 3.6 samples, Nocodazole (Merk Group, Darmstadt, Germany) 250 nm was added to culturing medium after 3 h from the seeding. After 24 h, samples were fixed and observed through Scanning Elecron Microscopy.

**Cell morphology.** Scanning Electron Microscopy (SEM) was used in combination with the orthogonal sample cutting through a Gallium Focused Ion Beam (FIB) source, to study cell morphology and their interactions with the underlying substrate. To perform the morphological assays 2 × 10^4^ MC3T3-E1, cells were plated on samples, and SEM-FIB preparation was performed 1, 6, 24, and 48 h after seeding at room temperature (RT). Briefly, culturing medium was removed, and cells were rinsed in PBS (Sigma-Aldrich, St. Louis, MI, USA). Subsequently, cells were fixed in a 2.5% glutaraldehyde solution (Sigma-Aldrich, St. Louis, MI, USA) prepared in Na-Cacodylate buffer (Sigma-Aldrich, St. Louis, MI, USA) for 30 min, thus washed in Na-Cacodylate buffer (Sigma-Aldrich, St. Louis, MI, USA) for 5 min, and dehydrated in ethanol at increasing concentrations (30%, 50%, 70%, 75%, 90%, 95%, and 99%) (Sigma-Aldrich, St. Louis, MI, USA). Finally, samples were critical point dried with liquid carbon dioxide (CPD 030 Baltec, Wallruf, Germany) and sputtered with a thin layer of gold through a SCD 040 coating device (Balzer Union, Wallruf, Germany). Sample microphotographs were taken using a dual beam Zeiss Auriga Compact system equipped with a GEMINI Field-Effect SEM column and a Gallium Focused Ion Beam (FIB) source (Zeiss, Oberkochen, Germany). SEM analysis was performed at 5 keV, while the cross-sectional analysis with FIB was performed with Gallium ion beam at 30 kV with a current of 10 pA.

A morphological analysis of cell perimeter, area and elongation factor was measured on SEM images at 6, 24, and 48 h of culture by Nikon Br5.11 software (Nikon, Tokyo, Japan). The elongation factor was obtained as the ratio of cell length (µm) to cell width (µm) in order to quantify the lengthening of cells on the different patterns.

**Cell viability assays.** Cell viability was assayed 24 and 48 h after plating with chemiluminescence assay (CellTiter Glo, Promega, Madison, WI, USA), according to the manufacturer’s indications. Briefly, culture medium was eliminated and a 50:50 solution of Cell-Titer Glo Lysis Buffer and serum free *α*-MEM was added to each sample. After 2 min of shaking, the solution was collected and luminescence was stabilized for 10 min in the dark. Subsequently, the luminescence was measured with a luminometer with double injectors (GLOMAX 20/20, Promega, Madison, WI, USA). Moreover, viable and dead cells were assessed 24 and 48 h after seeding by LIVE/DEAD imaging, which involved the use of Calcein-AM 4 µM (Sigma-Aldrich, St. Louis, MI, USA), specific for live cells and of Propidium Iodide 7.5 µM (Sigma-Aldrich, St. Louis, MI, USA), specific for dead cells. Briefly, culture medium was replaced with a PBS solution containing Calcein-AM and Propidium Iodide for 10min at RT in dark conditions. Subsequently, samples were washed in PBS and fixed for 20 min in paraformaldehyde 4% (PFA, Sigma Aldrich, St. Louis, MI, USA) and observed through fluorescence microscopy (Axioscope, Zeiss, Oberkochen, Germany).

**Immunofluorescence.** To observe expression and distribution of focal adhesions, an immunofluorescence staining was performed for focal adhesions, cytoskeleton, and cell nuclei. After 48 h of culture, culturing medium was removed, and cells were fixed in a 4% PFA solution for 10 min at RT. After two rinses in PBS, cells were permeabilized with 0.1% *v*/*v* Triton X-100 (Sigma-Aldrich, St. Louis, MI, USA) for 5 min at RT and washed twice with PBS. To block antibody aspecific binding sites, 1% Bovine Serum Albumin solution (BSA, Sigma-Aldrich, St. Louis, MI, USA) was added to the samples for 30 min at RT. Cells were then treated with a primary anti-Vinculin monoclonal antibody, clone 7F9 (1:100 dilution—FAK100, Merck Millipore, Darmstadt, Germany), for 1 h at RT and subsequently washed twice in PBS. To reveal primary anti-vinculin antibody, a secondary anti-mouse FITC-labeled (1:200 dilution—Thermo Fisher Scientific, Carlsbad, CA, USA) was co-incubated with TRITC (tetramethylrhodamine)-conjugated phalloidin (1:200 dilution—FAK100, Merck Millipore, Darmstadt, Germany) for actin staining. After three rinses with PBS, nuclei were counterstained with DAPI (4′,6-diamidino-2-fenilindol) solution (1:1000 dilution—FAK100, Merck Millipore, Darmstadt, Germany). Samples were observed and images were taken with a stereomicroscope equipped for fluorescence (SMZ25, Nikon, Tokyo, Japan).

**Statistical analysis.** Data were analyzed using Prism 7 (GraphPad, La Jolla, CA, USA). All values are reported as the mean ± standard deviation of three repeated experiments performed in triplicate. Differences between group means were evaluated with either *t*-Test or one-way ANOVA statistical test and Tukey post-test (cell viability quantification), and differences were considered significant when *p* < 0.05.

## 3. Results

### 3.1. SEM Morphology

**Figure 1:**
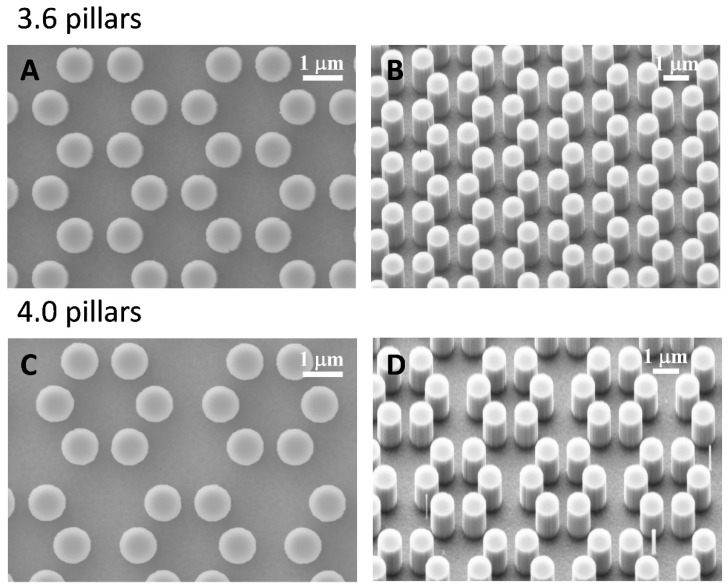
SEM microphotographs of 3.6 surfaces (**A**,**B**) and 4.0 (**C**,**D**) pillared samples.

We tested murine calvaria MC3T3-E1 cells on two kind of surfaces. The formers were represented by 0.9 µm high cylindrical pillars clustered in hexagons as shown in Figure 1. Surface wettability did not differ between 3.6 and 4.0 groups (data not shown).

**Figure 2:**
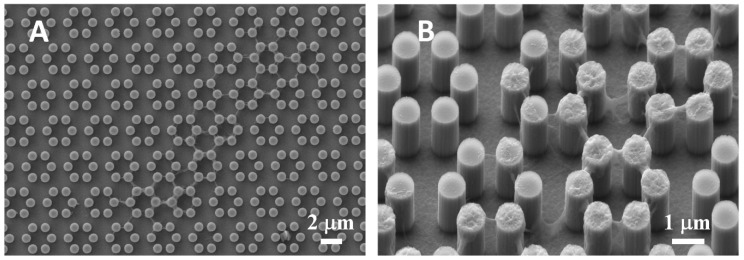
SEM microphotograph (**A**) of a 4.0 sample after incubation with complete culture medium, in the absence of cells. Protein precipitates are clearly visible on and between neighboring pillars in the enlargement (**B**).

Interestingly, when samples were pre-incubated for 24 h in complete medium, filamentous precipitates were observed connecting adjacent pillars (Figure 2). This finding was then observed more abundantly in the presence of cells, as expounded below.

**Figure 3:**
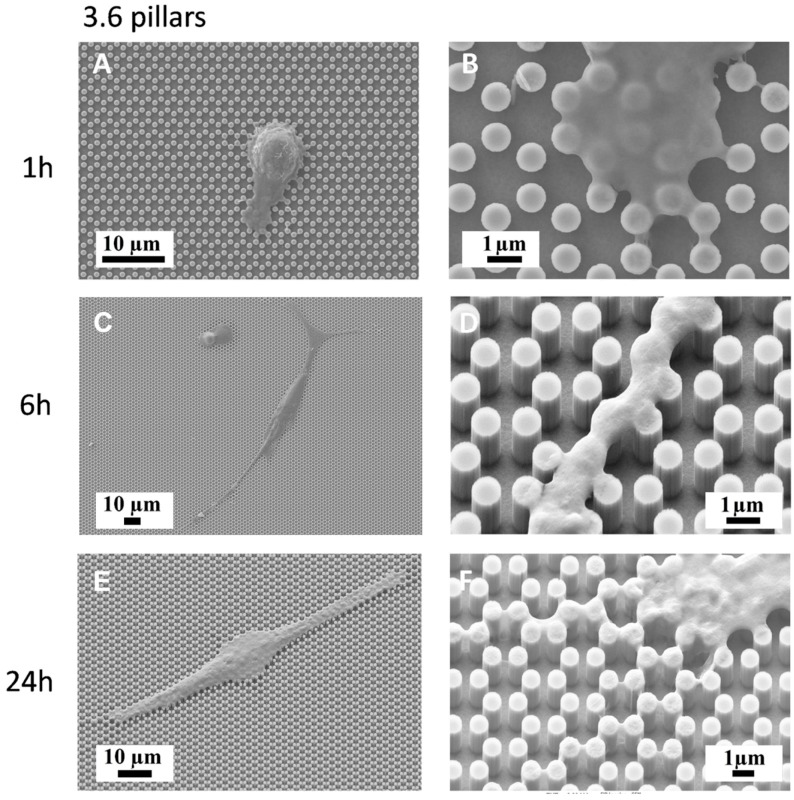
SEM microphotographs of MC3T3-E1 cells on 3.6 samples after 1 h of culture (**A**,**B**), 6 h of culture (**C**,**D**) or 24 h of culture (**E**,**F**).

**Figure 4:**
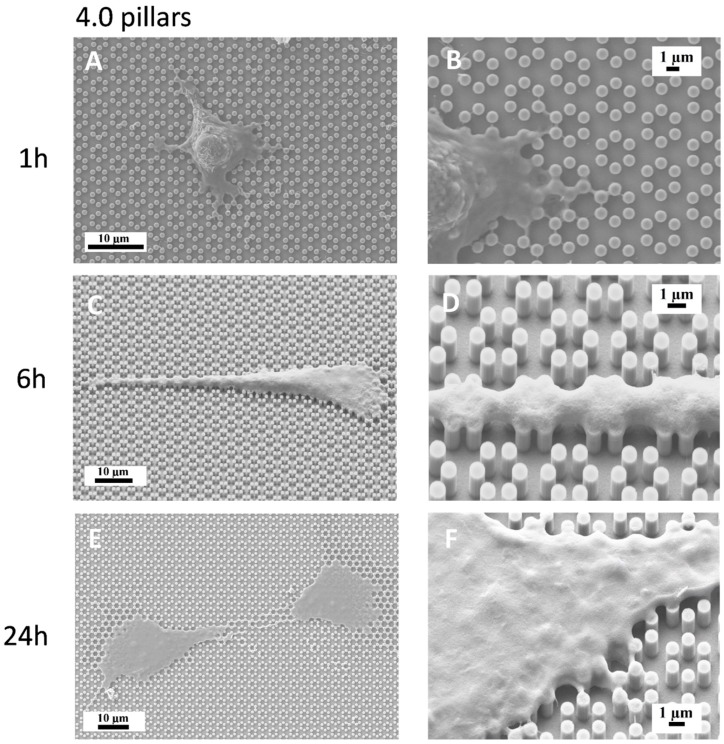
SEM microphotographs of MC3T3-E1 cells on 4.0 samples after 1 h of culture (**A**,**B**), 6 h of culture (**C**,**D**) or 24 h of culture (**E**,**F**).

**Figure 5:**
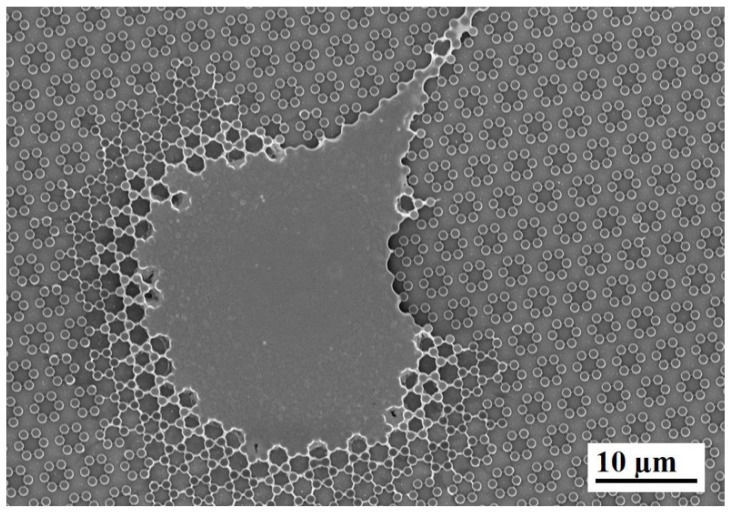
SEM microphotograph of a single cell on 4.0 samples after 24 h of culture; note the crown of inter-pillar filaments surrounding the whole leading edge.

**Figure 6:**
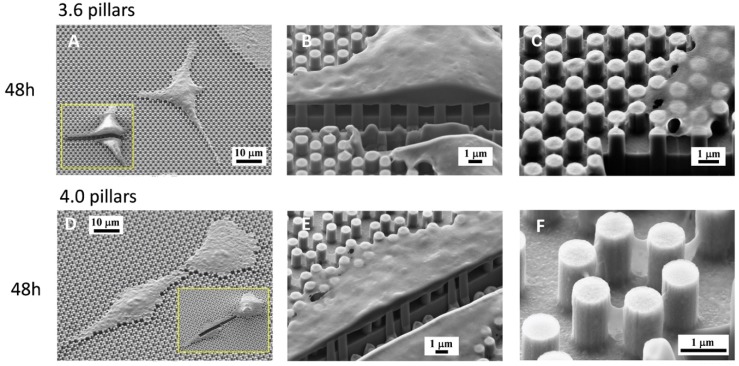
SEM microphotographs of osteoblastic cells on 3.6 (**A**–**C**) or 4.0 samples (**D**–**F**) after 48 h of culture. Enlargements of cells in (**B**,**C**,**E**) were sectioned by an FIB microscope.

We then plated MC3T3-E1 cells on both 3.6 and 4.0 surfaces and observed them with a SEM-FIB microscope after 1, 6, 24, and 48 h of culture (Figure 3, Figure 4, Figure 5 and Figure 6). After just 1 h of culture, cells displayed large lamellipodia that anchored and pulled the cell body by grasping onto groups of pillars or even whole pillar hexagons, while cells, as expected, maintained a rounder shape, especially on 3.6 samples (Figure 3A,B). Small, one-pillar projections were also visible around the cell body on 4.0 surfaces, as visible in Figure 4A,B.

By 6 h of culture, osteoblastic cells displayed elongated shapes, as they established their first cell-to-cell contacts, via long and thin projections, both on 3.6 and 4.0 surfaces (Figure 3C,D; Figure 4C,D). Interestingly cell membranes embraced not only the tip of the pillar but covered its sides, almost reaching its base.

At higher magnification, the microscope showed that uninterrupted chains of these septs departed from the cell body encasing groups of pillars already after 6 h and more markedly after 24 h of culture (Figure 3E,F; Figure 4E,F), like feelers exploring the extracellular environment (Figure 3F). These septs got to create a sort of aura around the whole cell body and joining adjacent cells and bridging over the gaps that separated neighbour cells. A remarkable example of these structures can be observed in Figure 5, which can be easily interpreted as representing a migrating cell, due to its distinctive morphology. The broader leading edge can be easily distinguished from the narrower rear edge (Figure 5). Most noticeably, a crown of inter-pillar filaments is seen surrounding the whole leading edge, while abruptly terminating on the rear end. At this time-point, several large one-hexagon-wide podosomes, which were not observed at earlier time-points, became visible around the cell body, beside lamellipodia (Figure 3F; Figure 4F).

FIB analysis of 3.6 and 4.0 surfaces at 48 h (Figure 6) showed, however, that the cell body, where intracellular tension was arguably higher, was stretched over the pillars, without entering the space in between the pillars (Figure 6B or Figure 6E), similarly to what we observed with SLA surfaces [20] and unlike the peripheral septs that can even cover whole pillars. The higher tension attained in the cytoplasm of the cell body is indirectly confirmed by its thinness, as the shape of the underlying pillars can be guessed through the cytoplasm (Figure 6C or Figure 6E). Only the nuclear area is obviously thicker at 48 h of culture, but not as thick as at 1 or 6 h and this implies a remarkable nuclear deformation, which the literature associated with a potent activator of differentiation [19]. FIB sections of 4.0 samples at 48 h (Figure 6E) suggest that the cell-to-pillar contact area may be somewhat larger than with 3.6 pillars (Figure 6B) and the cytoplasm can encase up to 20% of the height of the pillar (Figure 6E). Thin membrane projections that descend along the sides of the pillars are often undistinguishable from the precipitates described above (Figure 6F).

**Figure 7:**
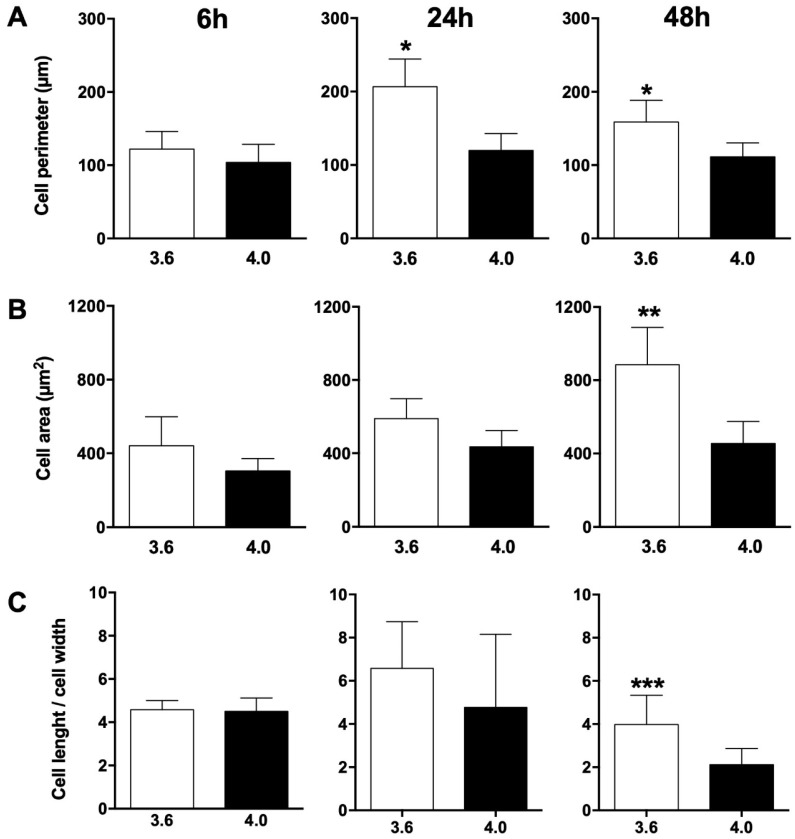
(**A**) cell perimeter, (**B**) area, and (**C**) elongation factor (as cell length/cell width ratio) of osteoblastic cells on 3.6 (white bars) or 4.0 (black bars) samples at 6, 24, or 48 h of culture. * *p* < 0.05 vs. 4.0 samples, ** *p* < 0.01 vs. 4.0 samples, *** *p* < 0.001 3.6 samples vs. 4.0 samples.

Cell morphology was analysed after SEM observation and cell perimeter, area and elongation factor were calculated at 6, 24, and 48 h of culture (Figure 7). Although no difference in cell perimeter was observed at 6 h of culture, cell perimeter on 3.6 surfaces was significantly higher than on 4.0 surfaces at both 24 (*p* = 0.0275) and 48 h (*p* = 0.0103) of culture (Figure 7A). Similarly, cell area on 3.6 surfaces was significantly greater than on 4.0 surfaces at 48 h of culture (*p* = 0.0018), while it only tended to be greater on 3.6 surfaces at 6 and 24 h of culture, albeit without reaching statistical significance (Figure 7B). Besides being bigger, cells on 3.6 surfaces were also more elongated, as shown in Figure 7C: the elongation factor (cell length/cell width) was significantly higher in cells on 3.6 surfaces, although only at 48 h (*p* = 0.0003).

### 3.2. Cell Viability

**Figure 8:**
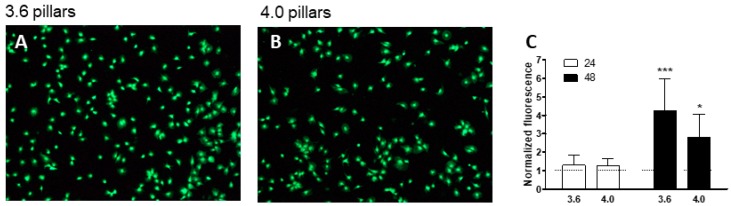
MC3T3-E1 cells after staining with Calcein AM (green) and Propidium iodide for cell viability observed at transmission optical microscope (**A**,**B**). Magnification = 20×. Cell viability was quantitated and expressed as a bar chart (**C**). *** *p* < 0.001 and * *p* < 0.05 vs. 24 h.

We assessed cell viability on 3.6 or 4.0 pillars by staining cells with vital dye Calcein-AM or Propidium Iodide, which stains dead cells (Figure 8A,B). Both surfaces confirmed supporting cell viability, as cells mostly presented green. We then quantitated fluorescence using a dedicated software (Nikon Br5.11 software, Nikon, Tokyo, Japan), as an index of cell viability, as presented in Figure 8C, normalized by the fluorescence levels on smooth silicon, used as control: cell viability at 24 h of culture was comparable to control, while cells on 3.6 pillars tended to display a higher level of viability by 48 h (Figure 8C). Cell viability in both groups at 48 h was higher than at 24 h (*p* < 0.001 in the 3.6 group and *p* < 0.05 in the 4.0 group).

### 3.3. Immunofluorescence

**Figure 9:**
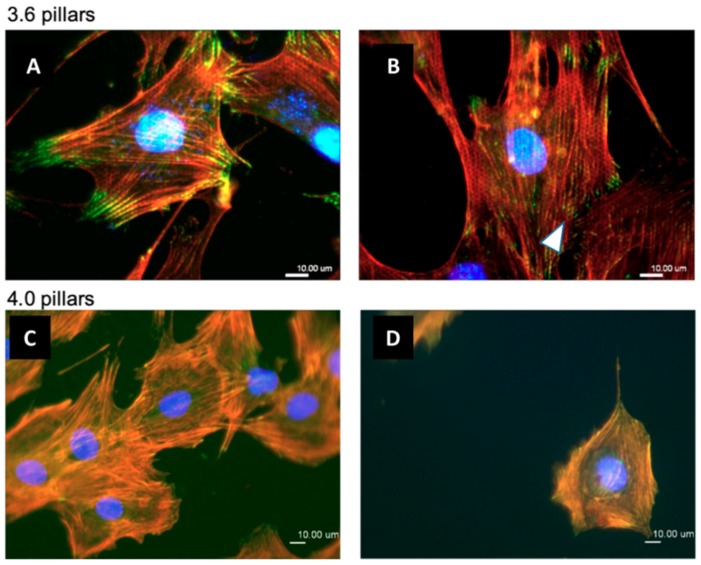
Immunofluorescence staining of osteoblastic cells on 3.6 samples (**A**,**B**) or 4.0 samples (**C**,**D**) after 24 of culture. Cell nuclei were stained with DAPI (blue), vinculin was stained with a FITC-labelled antibody (green) and actin microfilaments were marked with TRITC-conjugated phalloidin (red). Vinculin staining reproduced the hexagonal pattern of the underlying pillars (white arrowhead).

Osteoblastic cells were then stained for vinculin, using a primary anti-Vinculin monoclonal antibody, actin microfilaments, using TRITC-labelled phalloidin, and nuclei, with DAPI (Figure 9), after 24 h of culture. The cytoskeleton appeared composed by well visible stress fibers running across the whole cell body and anchored to the substrate by long adhesion complexes at the opposite extremities and showing small dot-like focal adhesions scattered on the cell base, on both 3.6 (Figure 9A,B) and 4.0 (Figure 9C,D) substrates. Interestingly, such dot-like focal adhesions could be seen arranged in hexagonal clusters (Figure 9B arrowhead), likely corresponding to the pattern of the pillars below. By comparing the outline of these hexagons with the actin fibers it is apparent that most stress fibers are actually aligned to the arrays of the underlying pillars. Actin filaments formed visible reinforcements around the pillars (Figure 9B), thus creating a honeycomb-like pattern in the red fluorescence labelling. Single channel fluorescence images of cells microphotographs are available in supplementary materials (Figure S1).

### 3.4. Effect of Cytoskeletal Inhibitors on Cell Adhesion

**Figure 10:**
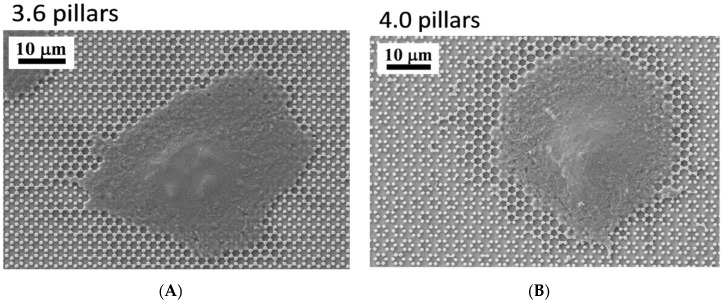
SEM microphotographs of osteoblastic cells on 3.6 (**A**) and 4.0 (**B**) samples after treatment with Nocodazole, an inhibitor of microtubule polymerization.

To investigate if changing the stability of the cytoskeleton would affect cell behaviour on pillars, we plated cells on the samples as described previously and treated them with 250 nM Nocodazole, a known inhibitor of microtubule polymerization. Cells, unsurprisingly, appeared mostly round, often flattened out on the surface and, interestingly, lost their prevalent orientation on the pillars (Figure 10). A vast distinctive aura of inter-pillar septs was visible all around the cells, both on 3.6 (Figure 10A) and on 4.0 substrates (Figure 10B). Furthermore, the cytoplasm showed signs of cell damage with increased porosity.

## 4. Discussion

The present study focuses on the adhesion of calvaria osteoblastic cells to micro-patterned surfaces provided with pillars arranged with two different inter-pillar spacings. The spatial arrangement of adjacent pillars created organized modules of hexagonal shape, which were clearly visible at SEM observation (Figure 1). Cells were seeded on these substrates and followed up as they adhered to the surfaces. Interestingly, they exhibited a strikingly similar behavior. One hour after seeding, cells—expectedly—were mostly round, with few broad projections embracing whole hexagons or even groups of hexagons (Figure 3A,B; Figure 4A,B), but, as time proceeded, the morphology of such projections and of the whole cell body changed quite distinctively. The cell body flattened out on the surface, as it is commonly observed even just on any plastic culture substrate, and the cytoplasmic projections became longer and thinner, after just 6 h of culture. Although cells as a whole still looked quite haphazardly arranged on the substrate, their projections followed the alignment of the underlying pillars, as if minimizing the inter-pillar gaps they had to bridge in order to attach. A careful observation of the contour of the cell body revealed, however, the presence of small hexagonal podosomes, or, rather, of small hexagonal cytoplasmic domains (Figure 3F; Figure 4F), indicating that the cytoplasm was still grasping onto whole pillar hexagons. When looked at immunofluorescence staining for vinculin, a protein that is found in focal adhesions (Figure 9B) [24], these cytoplasmic projections showed a high signal intensity, indicating that they are rich in focal adhesions. These focal adhesions actually closely matched the arrangement of the hexagons (Figure 9B, arrowhead), suggesting that these whole pillar hexagons, and not a part thereof, served as adhesion modules for the cells, similarly to what shown by Matschegewski et al. [25]. It is possible that adhering to a whole hexagon provided a mechanically firmer attachment than what would be obtained by attaching to only a fraction of the pillars therein. A simple hypothesis that can be put forth to explain this finding is that, by following the outline of the hexagons, cells can minimize the amount of cytoplasm that is hanging free in between adjacent pillars, as the side of a hexagon is geometrically shorter than its diagonal. This way, the distance between neighbouring focal adhesions is minimized as well. Cells therefore appeared to be able to attach to pillared or otherwise roughened surfaces quite effectively—so effectively actually to be able to fully differentiate on such surfaces, as extensively showed in the literature [26,27,28]—but always try to find out ways to minimize the cytoplasmic bridges between the irregularities, and appearing to prefer instead a *locum minoris distantiae*. This capability of discerning the geometric features of the substrate has been investigated in depth, and it has been shown that it requires cytoskeletal contractility and the activation of the RhoA/ROCK signalling pathway [29], and it is actually abolished by inhibiting actomyosin-generated tension [30,31]. Focal adhesion-mediated attachment is, however, necessary for cells to contract and thus recognize the pattern of the substrate [32]: adhesion is therefore central to cell responses to the substrate. Consistently with this idea, when cells on 4.0 surfaces were observed with the FIB microscope and sectioned, their grasp appeared deeper than on 3.6 pillars (Figure 6) and, since pillar size was unchanged on the two kinds of surfaces, this might just be a way to increase cell adhesion on the single pillar and thus compensate for longer inter-pillar bridging. This is in agreement with evidence indicating that osteoblasts attempt to internalize micropillars through a caveolin-mediated mechanism, to increase cell-to-substrate contact [33]. If cells strive to increase adhesion while minimizing hanging cytoplasm, it is not then maybe all that surprising that cells appeared more elongated on 3.6 than 4.0 surfaces (Figure 7), although it would probably be reasonable to expect cells to be more stretched on samples with a greater distance between hexagons. Cells, however, appear to be avoiding over-elongation and may end up stopping a few pillars shorter than they would if the next hexagon were in fact closer, as on 3.6 samples. These results are actually consistent with findings by Hasturk et al. [34]. Somewhat in agreement with this, the cell viability assay showed that cells tended to grow better on 3.6 surfaces than on 4.0 samples at 48 h of culture (Figure 8C), although they adhered equally well by 24 h, i.e., it was not a matter of how cells attached but rather of how they grew once they attached. Interestingly, however, cells grew better on patterned surfaces than on flat samples (Figure 8C), which is consistent with the literature indicating that there is a preferred pillar dimension and arrangement that is beneficial to osteoblasts [35].

Another striking finding is the presence of septs hanging in between pillars around cells and in direct contact with cells themselves. These could be theoretically interpreted in two alternative ways. They could be thin layers of cell membrane resulting from cell migration. In other words, for instance, it could be hypothesized that cells attach to the pillars and, as they creep along the surface by gripping on the pillars, they leave behind small fragments of cell membrane, which remain attached to the pillars, devoid of organelles or cytoskeleton, as these septs do not light up after immunofluorescence staining for microfilaments and vinculin. However, the available evidence goes strongly against this idea. When samples were incubated with complete culture medium and in the absence of cells, small deposits were observed at SEM (Figure 2), which can be explained as filamentous serum proteins, e.g., fibrinogen and/or fibronectin, precipitating on the pillars, similarly to in vitro fibronectin fibrillogenesis on polydimethylsiloxane pillars [36]. The structures we observed around cells then could have a similar origin and simply indicate protein precipitates, an aspect that will need to be investigated at a deeper level. Even more suggestively, their arrangement looks far from random (Figure 3F; Figure 4D). Figure 5 depicts a migrating cell, and these thin septs are visible only around the leading edge, while abruptly stopping at the rear, retracting end of the cell. This further observation rules out the possibility that they are cell remnants. The best explanation is therefore that they result from the selective secretion of proteins at the leading edge of the cells, which precedes cell movement. This would be consistent with findings by Schmoranzer et al. [37] who demonstrated an increase in the density of post-Golgi vesicles at the leading edge of migrating fibroblasts. If this is true, these proteins are creating ‘rails’ on which the cell body will attach, grasp on and progress. Figure 3F clearly indicates that these protein precipitates avoid the diagonals of hexagons too, possibly because the distance across hexagons is too wide to be bridged by protein auto-assembly alone. Cells then might be simply following the path outlined by proteins before they even attach to a pillar, and this would reinforce their preference for particular directions across pillars, as mentioned before. The immunofluorescence staining for actin filaments indicated that stress fibers within cells followed the main and preferred directions of the pillars along the same paths as the inter-pillar septs described above (Figure 9). When looking at higher magnification, actin forms rings around the pillars, in agreement with the experimental literature and models developed to explain it [25,38]. This actin conformation has the purpose of increasing the strength of cell adhesion to the substrate, as it has been shown that, when cell adhesion is confined to the top of pillars, cells generate significantly less contraction [39]. In agreement with our assumptions, microtubule impairment resulted in cells losing their orientation on the pillars (Figure 10) and the secretion of a large amount of proteins, which deposited around cells. This protein crown, which completely surrounded the cell bodies was not oriented as with normal cells, indicating that cells had changed their migration pattern, as microtubules failed to polymerize. Our results are consistent with previous reports showing that cells treated with Nocodazole were still able to move, although their migration was more random [40]. This might be associated with a random secretion of ECM proteins around the cell, as we observed in our samples, because microtubules have long been known to affect vescicular trafficking [41] and even caveolar trafficking [42], and their disruption might hamper the directional deposition of ECM on the pillars.

## 5. Conclusions

Taken together, these data suggest that cells condition the substrate around them by secreting extracellular components, likely of a protein nature. The deposition of these components is restrained, at least on such patterned surfaces, by the geometric conformation of the substrate material. The spatial arrangement of these precipitates matches the inner cytoskeletal organization and the alignment of cells on the substrate and all these elements, both precipitates and cell projections, seem to prefer pathways that minimize the gaps between irregularities. Future studies will have to address the nature of these precipitates and whether the substrate features affect the composition of such precipitates. This could be an important clue that could be easily tuned to control cell arrangement on the substrate, and, more importantly, possibly their behavior.

## Supplementary Materials

The following are available online at https://www.mdpi.com/2079-4991/9/12/1701/s1, Figure S1: Single channel immunofluorescence images of osteoblastic cells on 3.6 samples or 4.0 samples.
