# Supplementary material for: Sub-Micropillar Spacing Modulates the Spatial Arrangement of Mouse MC3T3-E1 Osteoblastic Cells
Source: Nanomaterials (Basel). 2019 Nov 28;9(12):1701. doi: 10.3390/nano9121701 (PMC6955749; doi:10.3390/nano9121701)
Supplement: Supplementary file 1 [file nanomaterials-09-01701-s001.pdf]

nanomaterials

Supplementary materials: Sub-Micropillar Spacing Modulates the Spatial Arrangement of Mouse MC3T3-E1 Osteoblastic Cells

Benedetta Ghezzi [1,2,*,†], Paola Lagonegro [3,4,†], Naoki Fukata [5,6], Ludovica Parisi [1,2,7], Davide Calestani [4], Carlo Galli [2], Giancarlo Salviati [4], Guido M. Macaluso [1,2,4] and Francesca Rossi [4]

1 Centro Universitario di Odontoiatria, Università di Parma, Via Gramsci 14, 43126 Parma, Italy; ludovica.parisi@unipr.it (L.P.); guidomaria.macaluso@unipr.it (G.M.M.)
2 Dipartimento di Medicina e Chirurgia, Università di Parma, Via Gramsci 14, 43126 Parma, Italy; carlo.galli1@unipr.it
3 ISMAC-CNR, Institute for macromolecular studies, Via Corti, 12, 20133 Milano, Italy; paola.lagonegro@gmail.com
4 IMEM-CNR, Institute of Materials for Electronics and Magnetism, Parco Area delle Scienze, 37/A, 43124 Parma, Italy; davide.calestani@imem.cnr.it (D.C.); giancarlo.salviati@cnr.it (G.S.); francesca.rossi@imem.cnr.it (F.R.)
5 International Center for Materials Nanoarchitectonics, National Institute for Materials Science, 1-1 Namiki, Tsukuba 305-0044, Japan; FUKATA.Naoki@nims.go.jp
6 Institute of Applied Physics, University of Tsukuba, 1-1-1 Tennodai, Tsukuba 305-8573, Japan
7 Labör für Orale Molekularbiologie, Klinik für Kieferorthopädie, Zahnmedizinische Klinik, Universität Bern, Freiburgstrasse 7, 3008 Bern, Switzerland
* Correspondence: benedetta.ghezzi@unipr.it
† These authors contributed equally to this work.

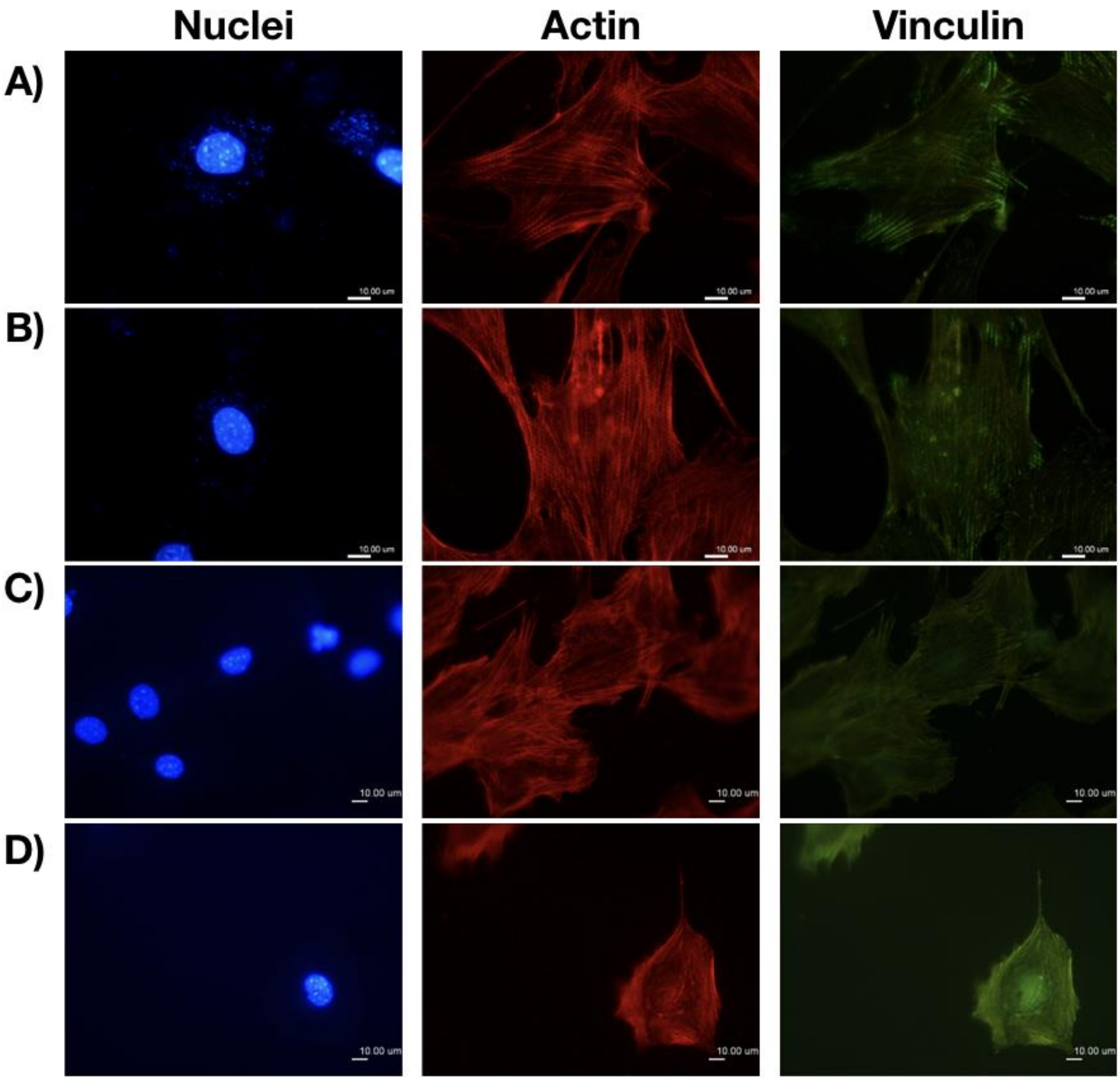

Figure S1. Single channel immunofluorescence images of osteoblastic cells on 3.6 samples (**A**–**B**) or 4.0 samples (**C**–**D**) after 24 hours of culture. Cell nuclei were stained with DAPI (blue), vinculin was stained with a FITC-labelled antibody (green) and actin microfilaments were marked with TRITC-conjugated phalloidin (red).
